# Exploring Advanced Practice Physiotherapist Scope of Practice by Discharge Diagnosis: A Review of 10 Years of Data From a Tertiary Hospital Emergency Department

**DOI:** 10.1111/1742-6723.70241

**Published:** 2026-03-08

**Authors:** Piers Truter, Louise Giglia‐Smith, Luke Bongiascia, Andrew Moffat, James Wrightson, Susan Brooker, Paul Atkinson, Pippa Flanagan

**Affiliations:** ^1^ School of Health Sciences, University of Notre Dame Australia Fremantle Western Australia Australia; ^2^ Physiotherapy Department Fiona Stanley Hospital Murdoch Western Australia Australia; ^3^ Physiotherapy Department Rockingham General Hospital Cooloongup Western Australia Australia; ^4^ School of Allied Health, Curtin University Bentley Western Australia Australia; ^5^ Physiotherapy Department Sir Charles Gairdner Hospital Nedlands Western Australia Australia; ^6^ Emergency Department Fiona Stanley Hospital Murdoch Western Australia Australia; ^7^ Department of Physical Therapy University of British Columbia Vancouver Canada; ^8^ Physiotherapy Department St John of God Midland Public Hospital Midland Western Australia Australia

**Keywords:** diagnosis, emergency department, musculoskeletal, physiotherapist, scope of practice

## Abstract

**Background:**

Musculoskeletal conditions are the most common low acuity ED presentation. In response, over the last 13 years, Advanced Practice Physiotherapist (APP) roles have been established in emergency departments (ED) across Australia. Despite APP roles being well established in many Australian EDs, the current scope of practice for APPs as defined by discharge diagnosis is not accurately defined.

**Objective:**

To derive a scope of practice based on ED diagnosis for APPs in an Australian tertiary hospital ED.

**Methods:**

Descriptive classification study of patients who received care from an APP in the study ED between January 2015 and September 2024, with an iterative consensus process to establish the scope of APP practice by ED discharge diagnosis.

**Results:**

APPs provided 37,771 individual episodes of care with 388 assigned International Classification of Diseases (10th revision) (ICD‐10) discharge diagnoses. 204 ICD‐10 diagnoses were rated as representing the local scope of practice for ED APPs. These codes accounted for 37,000 (98.2%) of the episodes of care. The APP team provided care for 33,713 adult patients, 4058 paediatric patients, 1197 patients who arrived by ambulance and 3477 patients referred to the ED by a GP. The consensus process demonstrated that APPs have clarity on their scope of practice and the capability to manage a differential diagnosis process that includes non‐musculoskeletal diagnoses in the ED setting.

**Conclusion:**

Using 10 years of ED data, a consensus process mapped the local scope of practice of APPs in a single Western Australian Tertiary Hospital ED to 204 ICD‐10 diagnoses.

## 1 Introduction

Globally, emergency departments (EDs) are under increasing pressure from high patient demand, including a high prevalence of low acuity musculoskeletal complaints. These patients often self‐refer and self‐transport to the ED and usually do not require hospital admission [1]. The high prevalence of this patient type has driven the development of primary contact, ED roles for physiotherapists, who are experts in the assessment, diagnosis and treatment of musculoskeletal conditions [2]. Advanced Practice Physiotherapists (APPs) are highly experienced, often with post‐graduate clinical qualifications [2, 3]. They undergo additional ED‐specific training, supervision and mentoring to develop the competencies needed for autonomous ED practice [2] and have become an embedded role in many Australian EDs.

Advanced Practice Physiotherapists have demonstrated high quality and safe patient care [2, 4, 5] while improving departmental efficiency [2, 6, 7] and reducing costs [2]. Patients report high levels of satisfaction with ED care provided by APPs, acknowledging their expertise in managing musculoskeletal conditions [2, 8, 9]. Advanced Practice Physiotherapists are also well received by ED staff; however, one commonly expressed staff concern relates to understanding of the APP role in ED, particularly around scope of practice [2, 8, 10]. This concern is focused on ED pressures to see as many patients as possible and perceived limitations of APPs who work within a musculoskeletal scope of practice [2]. Compared with doctors and nurse practitioners, this narrower scope also raises concerns for ED administrators regarding the number of ‘in scope’ patients that can be treated by an APP.

Scope of practice for APPs has previously been defined by urgency, age and diagnosis. APP scope as defined by urgency is commonly reported as Australasian Triage Scale (ATS) 3–5 or international equivalent [2, 3, 7, 11], and ATS 2 when co‐managed with a senior ED doctor [12] or streamed to ‘Fast Track’ [3]. In scope age ranges are reported as 8–65 [12], 18–65 [6], 18–80 [11] and any age as long as streamed to ‘Fast Track’ [3]. In terms of discharge diagnosis, APP scope of practice has previously been defined using 444 International Classification of Diseases (10th revision) (ICD‐10) diagnostic codes by an emergency physician establishing a research cohort [4], and 219 ICD‐10 diagnostic codes from all patients seen by APPs over 14 months in a tertiary hospital [2].

While these ICD‐10 lists provide insight into APP scope of practice, they included diagnoses such as fractured tooth, headache, migraine and thrombophlebitis, which are not traditionally associated with physiotherapy practice [2]. Despite advanced practice roles being well established in many Australian EDs, the current scope of practice for APPs as defined by discharge diagnosis is not accurately defined.

Therefore, our research aim was:

To derive a scope of practice based on emergency department diagnosis for APPs in a single Western Australian tertiary hospital ED.

## 2 Methods

### 2.1 Study Design

This was a descriptive classification study of episodes of care provided by an APP in the study hospital ED, with an iterative consensus process to establish scope of APP practice by ED discharge diagnosis. The study has been reported with consideration of the CREDES criteria [13].

### 2.2 Setting

The study ED is attached to a tertiary hospital in metropolitan Perth, Western Australia and had 111,145 presentations in 2023/24. There are separate children's and adult assessment and treatment areas. The adult area includes a Fast Track for lower acuity ambulant patients. The ED opened in 2015 with an APP workforce in the Fast Track multidisciplinary team. The APP team also provides care to patients in the children's area as they have capacity. During the study period, the APP team was rostered 7 days a week between the hours of 0800 and 2030. The APP autonomous scope of practice includes patients with musculoskeletal presentations, triaged 3–5 and aged 5–80. The APP team can select patients from the waiting room or ambulance bay, including patients referred to the ED by general practitioners (GP). The APPs independently order X‐rays of the limbs, assess, diagnose and manage patients to discharge from the ED. The APPs are not non‐medical prescribers. The study period was January 2015 to September 2024.

### 2.3 Participants

Data from episodes of care with an APP registered on the Emergency Department Information System (EDIS) as their ‘treating doctor’ were included.

### 2.4 Assignment of Diagnostic Codes to Patient Episodes

At the end of each episode of care, a discharge diagnosis is assigned to each episode, usually by the treating clinician or a senior doctor involved in patient care. Patients cannot be discharged from EDIS without a diagnosis, and occasionally this will be entered by another member of the ED team to remove a discharged patient from the ED active patient list. Diagnoses are presented on a ‘palette’ in EDIS, with a plain English description. While the palette description usually clearly matches a specific ICD‐10 code, there are some instances where there is some interpretation; for instance, T09.2 ‘Dislocation, sprain and strain of unspecified joint and ligament of trunk’ maps to the EDIS palette description of ‘Back muscle spasm’. For each episode of care, the most appropriate EDIS palette diagnosis is assigned to that episode.

### 2.5 Data Collection

Data were extracted from EDIS for included patients. The following variables were extracted: age at ED presentation (years); gender; Indigenous identification (dichotomised to Indigenous or other); referral source (dichotomised to referred by general practitioner (GP) or other); ATS [1, 2, 3, 4, 5]; arrival method (collapsed categories to private transport, ambulance, correctional services or other); time to be seen (min); length of stay (min); ICD‐10‐AM diagnosis code; and discharge disposition (collapsed categories to discharged, admitted or other). Date of ED presentation and Unique Medical Record Numbers (UMRN) were extracted to allow audit.

### 2.6 Iterative Consensus Process

The initial expert consensus panel was made up of five APPs with various amounts of clinical experience. Three have worked in the study ED since it opened (PF, LGS and PT), with over 10 years of ED experience. The other two APPs had 7 years (SB) and 2 years (PA) ED experience respectively. A mixture of experience levels was considered important as it matched the ED experience of the overall APP team in the study ED. This group of APPs was also chosen for their clinical experience across different ED settings. All APPs had clinical experience in at least two different EDs. Four APPs (PT, SB, PA, LGS) had experience from EDs in different health service districts in Perth and one APP (PT) had clinical experience from four EDs, including a Tertiary hospital ED in Queensland. This panel was considered appropriate because it included individuals with lived clinical experience in selecting patients for management as an APP, based on the presenting complaint and how this might link to discharge diagnosis, the potential differential diagnoses and the variability in presentation complexity.

The panel developed the scope of practice ratings shown in Table 1 and aimed for unanimous categorisation of each ICD‐10 code into one of the four codes. Where this was not possible or where it was unclear what cases a diagnosis represented (e.g., M79.89 Other specified soft tissue disorders, site unspecified), a medical record audit was assigned to one panel member. That member would report back to the panel on the audit findings. The iterations and voting continued until all ICD‐10 codes had at least three panellists in agreement.

**TABLE 1:** Scope of practice ratings for ICD‐10 codes.

Rating	Definition	Scope	Overall data
Core practice	Diagnosis suitable for independent management by an APP	Consensus scope	All associated ICD‐10 codes
Supported practice	Diagnosis suitable for APP to co‐manage with a senior ED doctor		
Fringe practice	Not a diagnosis that APPs would currently seek to manage, but possible future scope expansion	Excluded from scope	
Not suitable	Diagnosis not suitable for management by an APP		

An emergency physician (AM) with 8 years of ED experience and significant experience working with APPs in the study was included in the final iteration to provide an alternative non‐APP perspective and additional validation.

#### 2.6.1 Iteration One

The consensus panel was provided with a list of all ICD‐10 diagnosis codes associated with episodes of care managed by the APP team in the ED during the study period (group labelled ‘all associated codes’). The panel were asked to independently rate each ICD‐10 code against the ratings shown in Table 1. Recognising the variety in patient presentations that each diagnosis represents, panellists were asked to choose the rating that most patients (> 80%) with that diagnosis would be associated with.

#### 2.6.2 Iterations Two to Four

Panellists were presented with the independent voting and audit information from the previous iteration. Where there was unanimous agreement, no further voting was required. Where further information was required on patient clinical characteristics, further medical record audits were conducted and reported back. Panellists then voted again on diagnoses without complete agreement.

#### 2.6.3 Iteration Five

The first author (PT) reviewed all associated ICD‐10 codes, votes and audit outcomes to make a recommendation to the panel on each rating. The recommendations were presented to the panel, who endorsed a preliminary decision on scope of practice rating (Table 1) for all ICD‐10 codes.

#### 2.6.4 Iteration Six—ED Physician Added to the Panel

The initial panel members met with an emergency physician (AM) to review and endorse the ICD‐10 code ratings. This produced the final consensus scope ICD‐10 group (group labelled ‘consensus scope’), made up of the core and supported practice rated ICD‐10 codes (Table 1), as these were felt to represent the contemporary practice of the APPs in the study ED.

### 2.7 Data Analysis

The EDIS variables were reviewed and length of stay variables for 2 patients (197,459 and 43,276 min) were replaced with the next highest value (1991 min), as the values appeared to represent error.

Descriptive statistics were generated for the ‘all associated codes’ and ‘consensus scope’ groups. The consensus panel identified three important sub‐groups in the consideration of APP scope of practice; (i) Paediatric patients, (ii) Patients arriving by ambulance and (iii) Patients referred to the ED by a GP. Descriptive statistics for the sub‐groups were generated from the adjusted scope group. Data was analysed using the Microsoft Excel (Microsoft 365 MSO [Version 2502 Build 16.0.18526.20546] 64‐bit).

Key themes from the consensus process are presented narratively.

### 2.8 Ethics

Approval for data collection and publication was received through registration with South Metropolitan Health Service quality improvement processes (GEKO 54948).

## 3 Results

### 3.1 Scope of Practice

The APP team provided 37,771 episodes of care with 388 assigned ICD‐10 diagnoses in the study period (Table 2). The consensus panel rated 34 of these diagnoses as fringe practice, and 150 as not suitable. These 184 diagnoses were excluded (total of 771 patients). The remaining 204 ICD‐10 diagnoses (185 core practice and 19 supported practice) are defined as the consensus scope. These account for 37,000 (98.2%) of all associated ICD‐10 codes (Table 2). Within the consensus scope, the APP team provided care for 32,942 adult patients, 4058 paediatric patients, 1197 patients who arrived by ambulance, and 3477 patients referred to the ED by a GP. The demographics for these sub‐groups are shown in Table 2. The full list of ICD‐10 codes is in Supporting Information.

**TABLE 2:** Demographics of all associated codes and consensus scope groups, with consensus scope sub‐groups for paediatric patients, patients arriving by ambulance and patients referred by GP.

	All associated codes	Consensus scope	Paediatric patients	Ambulance arrivals	GP referrals
Number of ICD‐10 codes	388	204	117	109	149
Number of episodes of care	37,771	37,000	4058	1197	3477
GP referrals	3541 (9%)	3477 (9%)	410 (10%)	15 (1%)	3477 (100%)
Age range (years)	1–98	1–98	1–15	7–98	2–90
Age (Med (IQR)) (years)	33 (21–49)	33 (21–49)	12 (10–14)	42 (27–58)	35 (22–53)
Paediatric patient (under 16 years)	4086 (11%)	4058 (11%)	4058 (100%)	28 (2%)	410 (12%)
Age bands (years)					
0–10	1154 (3%)	1147 (3%)	1147 (28%)	6 (1%)	143 (4%)
11–20	7904 (21%)	7805 (21%)	2911 (72%)	143 (12%)	635 (18%)
21–30	8254 (22%)	8081 (22%)	0 (0%)	218 (18%)	691 (20%)
31–40	6395 (17%)	6272 (17%)	0 (0%)	204 (17%)	566 (16%)
41–50	5513 (15%)	5382 (15%)	0 (0%)	185 (15%)	475 (14%)
51–60	4376 (12%)	4272 (12%)	0 (0%)	186 (16%)	423 (12%)
61–70	2779 (7%)	2705 (7%)	0 (0%)	138 (12%)	340 (10%)
71–80	1140 (3%)	1090 (3%)	0 (0%)	83 (7%)	169 (5%)
81–90	243 (1%)	233 (1%)	0 (0%)	29 (2%)	35 (1%)
91–100	13 (0%)	13 (0%)	0 (0%)	5 (0%)	0 (0%)
Gender (Female)	16,966 (45%)	16,645 (45%)	1691 (42%)	640 (53%)	1592 (46%)
Indigenous^a^	1496 (4%)	1328 (4%)	111 (3%)	68 (6%)	88 (3%)
Arrival method					
Private transport	35,892 (95%)	35,184 (95%)	4014 (99%)	0 (0%)	3446 (99%)
Ambulance	1246 (3%)	1197 (3%)	28 (1%)	1197 (100%)	15 (0%)
Correctional services	542 (1%)	533 (1%)	7 (0%)	0 (0%)	11 (0%)
Other	91 (0%)	86 (0%)	9 (0%)	0 (0%)	5 (0%)
Triage category (ATS)					
1—Resuscitation	2 (0%)	0 (0%)	0 (0%)	0 (0%)	0 (0%)
2—Emergency	252 (1%)	248 (1%)	14 (0%)	37 (3%)	7 (0%)
3—Urgent	4551 (12%)	4414 (12%)	309 (8%)	360 (30%)	287 (8%)
4—Semi‐urgent	26,125 (69%)	25,683 (69%)	2900 (71%)	756 (63%)	2390 (69%)
5—Non‐urgent	6841 (18%)	6655 (18%)	835 (21%)	44 (4%)	793 (23%)
Time to being seen (Med (IQR)) (min)	35 (19–63)	35 (19–63)	38 (21–66)	35 (17–73)	37 (20–64)
Length of stay (Med (IQR)) (min)	120 (88–165)	120 (88–164)	106 (78–143)	175 (128–233)	116 (84–163)
Care managed within 4 h	35,472 (94%)	34,934 (94%)	3955 (97%)	952 (80%)	3301 (95%)
Disposition					
Discharged	36,748 (97%)	36,147 (98%)	4005 (99%)	1037 (87%)	3396 (98%)
Admitted	784 (2%)	709 (2%)	42 (1%)	153 (13%)	65 (2%)
Other	239 (1%)	144 (0%)	11 (0%)	7 (1%)	16 (0%)

*Note:* Providing care for patients with traumatic injuries to the upper and lower limbs, made up 85% of the APP team consensus scope caseload and included 134 (65%) diagnoses. A caseload summary is shown in Table 3.

Abbreviations: ATS, Australasian Triage Score; IQR, Inter‐quartile range; Med, Median.

^a^ 139 missing values.

**TABLE 3:** Consensus scope caseload summary by clinical domains.

Clinical domain	Core scope		Supported practice		Consensus scope	
	No. ICD‐10 codes	No. of cases	No. ICD‐10 codes	No. of cases	No. ICD‐10 codes	No. of cases
Atraumatic pain	28	1130	5	118	33	1248
Spinal pain	25	3162	2	5	27	3167
Upper limb trauma	65	14,218	4	91	69	14,309
Lower limb trauma	59	17,153	4	134	63	17,287
Total	185	36,553	19	447	204	37,000

The most common discharge diagnoses are shown in Table 4 for the consensus scope and the three sub‐groups of paediatric patients, patients arriving by ambulance and GP referred patients. These lists are comprised of traumatic limb injuries and low back pain diagnoses. In the consensus scope, 19 discharge diagnoses were rated as supported practice and are listed in Table 5.

**TABLE 4:** Most common 20 discharge diagnoses for consensus scope, paediatric patients, patients who arrived by ambulance and GP referred patients.

Consensus scope			Paediatric patients			Ambulance arrivals			GP referred patients		
ICD‐10	Diagnosis	Number	ICD‐10	Diagnosis	Number	ICD‐10	Diagnosis	Number	ICD‐10	Diagnosis	Number
S93.40	Ankle sprain	3876	S62.60	Finger fracture	453	S33.50	Strain of lumbar spine	137	S62.60	Finger fracture	278
S83.6	Knee sprain	2081	S93.40	Ankle sprain	421	S93.40	Ankle sprain	111	S92.20	Tarsal bone fracture	272
S92.20	Tarsal bone fracture	1808	S63.60	Finger sprain	272	S83.6	Knee sprain	80	S93.40	Ankle sprain	216
S62.60	Finger fracture	1533	S63.50	Wrist sprain	196	S83.0	Dislocation of patella	66	S92.9	Toe fracture	189
S92.9	Foot fracture	1215	S52.50	Distal radius fracture	177	M54.5	Low back pain	51	S82.6	Foot fracture	180
S82.6	Lateral malleolus fracture	1124	S92.9	Toe fracture	146	S82.6	Lateral malleolus fracture	48	S62.30	Lateral malleolus fracture	175
S33.50	Strain of lumbar spine	1111	S92.20	Foot fracture	145	S23.3	Tarsal bone fracture	30	S62.2	Metacarpal fracture	170
S62.30	Metacarpal fracture	1066	S82.6	Tarsal bone fracture	135	S92.20	Strain of the thoracic spine	30	S52.50	Distal radius fracture	129
S93.6	Foot sprain	1001	S62.50	Lateral malleolus fracture	131	S43.00	Shoulder dislocation	27	S83.6	Knee sprain	110
S63.50	Wrist sprain	993	S83.6	Thumb fracture	125	S82.0	Fracture of patella	27	S52.30	Shaft of radius fracture	89
S62.2	First metacarpal fracture	902	S93.6	Knee sprain	115	S82.18	Proximal tibial fracture	23	S62.50	Thumb fracture	87
S52.50	Distal radius fracture	869	S62.30	Foot sprain	110	S42.00	Clavicle fracture	22	S63.60	Finger sprain	57
S63.60	Finger sprain	868	S52.5	Metacarpal fracture	105	M19.99	Osteoarthritis	21	S52.00	Proximal ulna fracture	56
S60.2	Wrist or hand contusion	758	S52.30	Wrist or hand fracture	99	M54.19	Radiculopathy	21	S93.6	Foot sprain	55
S52.30	Shaft of radius fracture	610	S62.8	Shaft of radius fracture	99	S73.10	Shaft of tibia fracture	20	S63.50	Wrist sprain	52
S90.3	Foot contusion	597	S60.2	Wrist or hand contusion	94	S82.28	Hip sprain	20	S82.18	Injury of Achilles tendon	51
M54.5	Low back pain	563	S62.10	Carpal bone fracture	73	S83.2	Meniscus tear	20	S86.0	Proximal tibial fracture	51
S83.2	Meniscus tear	523	S60.0	Finger contusion	72	S52.50	Distal radius fracture	19	M54.19	Radiculopathy	47
S62.50	Thumb fracture	504	S42.00	Clavicle fracture	67	S82.81	Bimalleolar ankle fracture	18	S82.0	Fracture of patella	45
S43.7	Shoulder sprain	460	S52.00	Ulna fracture	66	S62.8	Wrist or hand fracture	17	S33.50	Strain of lumbar spine	43
Total		22,462			3101			808			2393

**TABLE 5:** Diagnoses coded to supported practice and number of patients with these diagnoses.

ICD‐10	Diagnosis	Number
S72.40	Fracture of lower end of femur	68
M10.99	Gout	56
S22.40	Rib fracture (s)	54
S82.82	Trimalleolar ankle fracture	53
S63.08	Wrist dislocation	40
M70.4	Prepatellar bursitis	33
M70.2	Ulna and radius shaft fracture	27
S52.4	Olecranon bursitis	27
S53.10	Elbow dislocation	21
T00.9	Multiple superficial injuries	19
S43.6	Sternoclavicular joint sprain	18
S93.30	Foot dislocation	10
S43.2	Sternoclavicular joint dislocation	8
S34.3	Ankle dislocation	3
S63.00	Wrist dislocation, other	3
S93.0	Injury of cauda equina	3
G95.2	Cord compression	2
M11.99	Crystal arthropathy	1
M91.9	Juvenile osteochondrosis of hip and pelvis	1
Total		447

### 3.2 Findings From the Consensus Process

Key discussion themes from the consensus process were:

#### 3.2.1 Understanding of Scope of Practice

APPs demonstrated a comprehensive understanding of their scope of practice, with in‐depth discussions on differential diagnosis processes that lead to a final diagnosis. APPs understood that a discharge diagnosis might be in‐scope, but that the differential diagnosis might require medical input.

#### 3.2.2 Differences Between APPs

There was agreement on core practice for APPs in ED, but variability in differential diagnostic complexity that individual APPs were comfortable managing. This represented differences in experience, practice in different EDs (all APPs had worked in EDs other than the study ED), and on‐the‐job exposure to diagnoses.

#### 3.2.3 Understanding of Variability Within a Diagnosis

All panellists acknowledged the wide variety of presentations that can contribute to a discharge diagnosis and that APPs also considered presenting complaint, acuity, comorbidity, red flags and treatment needs when selecting patients. This reflects that while many patients with a diagnosis may be suitable for care from an APP, others may need multi‐disciplinary care.

#### 3.2.4 Confirming Safe Practice by the APP Team

Although 184 diagnoses were labelled as ‘fringe practice’ or ‘not suitable’, this represented only 771 patients (1.8% of the audit), with all being managed safely within the defined scope of practice. Audit of these excluded diagnoses showed these were largely due to EDIS data entry errors, inconsistent diagnostic coding, and APPs working in a multidisciplinary ED team alongside senior doctors for out‐of‐scope patients after handover.

## 4 Discussion

One defining dimension of APP scope of practice was developed using discharge diagnoses from 10 years of episodes of care managed by APPs in a single Australian tertiary ED. This comprised 204 ICD‐10 diagnosis codes representing the diagnosis‐based profile of local practice of APPs in the study ED. The consensus process highlighted a shared clarity on the boundaries of APP scope of practice, including a differential diagnosis process that recognises non‐musculoskeletal diagnoses for apparent musculoskeletal presentations. Working within scope and with the multi‐disciplinary team in the ED is important because a credentialled APP can select, manage and discharge patients from the ED independently [14]. This study has highlighted the value of APPs working within the multi‐disciplinary ED team both independently within their core practice and alongside medical colleagues for conditions requiring supported practice.

APP scope has been quantified previously for research purposes, by ICD‐9 diagnosis by a single emergency physician, in order to define a comparison cohort for a new ED APP service [4]. Compared to the consensus scope ICD‐10 list established in this study, there is broad overlap of many in‐scope diagnoses, however some diagnoses, including dislocation of large joints (e.g., shoulder), were omitted. Thompson, Williams [2] report a list of ICD‐9/ICD‐10 diagnoses for patients who were managed by APPs in a Tertiary Hospital ED in South Australia over a pilot period of 14 months. This list also appears similar to the consensus scope ICD‐10 list but contains diagnoses considered out of scope in this study (e.g., headache). There is agreement that APP's primary caseload includes traumatic peripheral injuries of the upper and lower limbs, and low back pain [3, 4, 12]. At the study ED, the APP team provided care to children, patients arriving by ambulance and patients referred by GPs. The APPs selected patients from a narrower scope of practice with these sub‐groups in terms of discharge diagnosis (e.g., paediatric patients, 117 ICD‐10 codes) and generally managed lower acuity patients likely to discharge home from the ED.

### 4.1 Limitations

This study was conducted through an iterative consensus approach. The study authors also made up the membership of the expert panel. As such, the ratings of each ICD‐10 diagnosis by each panel member were not concealed. The scope of practice ratings also reflect practice specific to the study ED, and it is acknowledged that APP credentialling and scope of practice are varied across sites, states and internationally.

Some of the ICD‐10 code variability was revealed through audit to be linked to different practices between APPs in applying diagnostic codes in EDIS. Some ICD‐10 codes may therefore be unique to the study ED. The assignment of an ICD‐10 code to an episode of care can also be by any member of the ED team and this can introduce error into the coding process. These factors may reduce the generalisability of the consensus scope ICD‐10 list. The consensus process was designed to make these issues transparent and to mitigate them where possible.

Finally, diagnosis only reflects one dimension of APP scope. Scope is also defined by age, presenting complaint, risk, comorbidity, red flags and urgency. It is also defined by the specific characteristics, needs and individual preferences of patients. This was a descriptive classification study that derived a diagnosis‐based profile of local APP practice and reflected that the APP panellists had a comprehensive understanding of scope and diagnostic reasoning. Further research is needed to establish generalisability to other ED settings, to develop a robust methodology to identify APP suitable caseloads in the ED and to investigate safety and effectiveness of APP clinical practice.

## 5 Conclusion

A consensus process, based on 10 years of ED data, mapped the scope of practice by APPs working in a Western Australian Tertiary Hospital ED to 204 ICD‐10 diagnoses. The consensus process demonstrated that APPs have clarity on their scope of practice and the capability to manage a differential diagnosis process that includes non‐musculoskeletal diagnoses in the ED setting.

## Funding

The authors have nothing to report.

## Ethics Statement

Approval for data collection and publication was received through registration with South Metropolitan Health Service quality improvement processes (GEKO 54948).

## Conflicts of Interest

The authors declare no conflicts of interest.

## Supporting information


**Data S1:** emm70241‐sup‐0001‐Supinfo.xlsx.

## Data Availability

The data that supports the findings of this study are available in the Supporting Information of this article.
